# Indirect Role of AQP4b and AQP4d Isoforms in Dynamics of Astrocyte Volume and Orthogonal Arrays of Particles

**DOI:** 10.3390/cells9030735

**Published:** 2020-03-17

**Authors:** Marjeta Lisjak, Maja Potokar, Robert Zorec, Jernej Jorgačevski

**Affiliations:** 1 Laboratory of Neuroendocrinology–Molecular Cell Physiology, Institute of Pathophysiology, Faculty of Medicine, University of Ljubljana, Zaloška 4, 1000 Ljubljana, Slovenia; marjeta.lisjak@mf.uni-lj.si (M.L.); maja.potokar@mf.uni-lj.si (M.P.); robert.zorec@mf.uni-lj.si (R.Z.); 2 Celica Biomedical, Tehnološki park 24, 1000 Ljubljana, Slovenia

**Keywords:** aquaporin 4 (AQP4), AQP4b, AQP4d, AQPe4, endosome, lysosome, Golgi apparatus, astrocyte, structured illumination microscopy, brain edema

## Abstract

Water channel aquaporin 4 (AQP4) plays a key role in the regulation of water homeostasis in the central nervous system (CNS). It is predominantly expressed in astrocytes lining blood–brain and blood–liquor boundaries. AQP4a (M1), AQP4c (M23), and AQP4e, present in the plasma membrane, participate in the cell volume regulation of astrocytes. The function of their splicing variants, AQP4b and AQP4d, predicted to be present in the cytoplasm, is unknown. We examined the cellular distribution of AQP4b and AQP4d in primary rat astrocytes and their role in cell volume regulation. The AQP4b and AQP4d isoforms exhibited extensive cytoplasmic localization in early and late endosomes/lysosomes and in the Golgi apparatus. Neither isoform localized to orthogonal arrays of particles (OAPs) in the plasma membrane. The overexpression of AQP4b and AQP4d isoforms in isoosmotic conditions reduced the density of OAPs; in hypoosmotic conditions, they remained absent from OAPs. In hypoosmotic conditions, the AQP4d isoform was significantly redistributed to early endosomes, which correlated with the increased trafficking of AQP4-laden vesicles. The overexpression of AQP4d facilitated the kinetics of cell swelling, without affecting the regulatory volume decrease. Therefore, although they reside in the cytoplasm, AQP4b and AQP4d isoforms may play an indirect role in astrocyte volume changes.

## 1. Introduction

Aquaporin-4 (AQP4) is a transmembrane channel that selectively transports water molecules and is expressed in different cell types throughout the body [1,2,3,4,5,6]. In the central nervous system (CNS), AQP4 is expressed in ependymal cells facing the ventricles, and in the end feet of astrocytes lined along synapses, the perivessel, and subpial brain areas [7,8,9,10]. Among all AQP types expressed in the CNS (AQP1, AQP4, and AQP9), AQP4 is the most abundantly expressed brain aquaporin, with a predominant expression in astrocytes [5,6]. In tissue, the distribution of AQP4 in astrocytes in certain parts of the CNS is clearly polarized, where AQP4 is primarily concentrated in the plasma membrane of astrocytic processes in close proximity to blood vessels, the ependymal layer, and pia [8,9]. In distinct osmosensory CNS areas, glial processes show little or even no polarization of the AQP4 distribution [8]; in rat and mouse astrocyte cultures, AQP4 is localized at the plasma membrane and intracellularly [11,12]. Initially, two AQP4 isoforms were described—AQP4a (M1) and AQP4c (M23)—which are alternative transcripts from two different initiating methionine sites [1,13,14,15,16,17]. A decade ago, four additional isoforms of AQP4 were described by screening the rat AQP4 gene, which encodes six isoforms [15]. The discovery of the additional isoforms AQP4b, AQP4d, AQP4e, and AQP4f, led to the introduction of a uniform terminology naming the AQP4 isoforms a–f, where AQP4a and AQP4c correspond to the two classic M1 and M23 isoforms, respectively [15]. Outside the CNS, another AQP4 isoform was described in human skeletal muscle—AQP4-Δ4—which is apparently exclusively intracellular [18]. Of the six AQP4 isoforms in the CNS, AQP4a, AQP4c, and AQP4e are water-permeable and have been shown to cluster into orthogonal arrays of particles (OAPs) in the plasma membrane of astrocytes and human skeletal muscles [19,20,21,22,23,24,25]. These three isoforms have been shown to affect the volume regulation in astrocytes [19,26], which are very sensitive to changes in extracellular osmolarity [27]. In hypoosmotic conditions, these cells swell rapidly and then correct for their volume excess by undergoing the process of a regulatory volume decrease (RVD) [28,29]. The swelling phase and the RVD have been shown to be modified by AQP4 expression in astrocytes, in particular, by the AQP4a, AQP4c, and AQP4e isoforms [19,26,30].

AQP4a, AQP4c, and AQP4e alternatively splice into AQP4b, AQP4d, and AQP4f isoforms, respectively. Their splicing variants, which lack exon 2, have only four transmembrane helices instead of six. This likely renders them incapable of water transport, as demonstrated in *Xenopus* oocytes [4,15]. It has been proposed that alternatively spliced isoforms of AQP4 may affect the translocation of water-permeable isoforms to the plasmalemma [18]; however, this hypothesis has not been tested yet for the CNS intracellular AQP4 isoforms. Moreover, the intracellular localization of AQP4b, AQP4d, and AQP4f in astrocytes has not yet been investigated. The aim of our study was to determine the cellular localization of AQP4b and AQP4d isoforms and to assess whether their expression can affect changes in the cell volume of rat astrocytes.

## 2. Materials and Methods

### 2.1. Cell Cultures

Cortical astrocytes were isolated from 2- to 3-day-old female Wistar rats by a standard procedure [12] and maintained in growth medium (high-glucose Dulbecco’s modified Eagle’s medium supplemented with 10% fetal bovine serum, 1 mM sodium pyruvate, 2 mM L-glutamine, and 25 μg/mL penicillin/streptomycin; Innoprot, Derio, Spain) at 37 °C in 95% air/5% CO_2_. Cells were plated onto 22-mm diameter 1% poly-L-lysine-coated coverslips and used within 4 days after plating. All chemicals were purchased from Sigma-Aldrich (Merck KGaA, Darmstadt, Germany), unless stated otherwise. The care of experimental animals and euthanasia of animals were performed in accordance with the following ethical codes and directives: International Guiding Principles for Biomedical Research Involving Animals developed by the Council for International Organizations of Medical Sciences and the Directive on Conditions for Issue of License for Animal Experiments for Scientific Research Purposes (Official Gazette of the Republic of Slovenia 40/85, 22/87, 43/07). The protocol for the euthanasia of animals used in our study was approved by the Veterinary Administration of the Ministry for Agriculture and the Environment of the Republic of Slovenia (permit no. 34401-29/2009/2), issued on 22 April 2009.

### 2.2. Hypoosmotic Stimulation

The extracellular isoosmotic solution (300 ± 10 mOsm) consisted of 130 mM NaCl, 5 mM KCl, 2 mM CaCl_2_, 1 mM MgCl_2_, 10 mM D-glucose, and 10 mM HEPES (pH 7.2). Hypoosmotic conditions (200 mOsm) were obtained by reducing the osmolarity of the isoosmolar medium from 300 to 200 mOsm by the application of 100 mOsm hypoosmotic solution. The hypoosmotic solution (100 ± 10 mOsm) consisted of 30 mM NaCl, 5 mM KCl, 2 mM CaCl_2_, 1 mM MgCl_2_, 10 mM D-glucose, and 10 mM HEPES (pH 7.2). The osmolarity of the solutions was measured with a freezing-point osmometer (Osmomat030, Gonotec, Berlin, Germany).

### 2.3. Aquaporin 4 Labeling and Immunocytochemistry

Recombinant AQP4b, AQP4d, and AQP4e isoforms were overexpressed by transfecting cells with pAQP4b-EGFP (AQP4b), pAQP4d-EGFP (AQP4d), and pAQP4e-EGFP (AQP4e) (a gift from O.P. Ottersen’s laboratory, University of Oslo, Oslo, Norway) using FuGENE 6 (Promega, Madison, WI, USA), according to the manufacturer’s instructions. Cells were used in the experiments 48–80 h after transfection.

OAPs were labeled with neuromyelitis optica (NMO)-IgG antibodies (autoantibodies formed in NMO patients) that recognize the extracellular epitope of AQP4 without affecting the OAP size or inducing AQP4 endocytosis [19,21,31,32,33,34]. Non-permeabilized cells were incubated in isoosmotic (300 mOsm) or hypoosmotic (200 mOsm) solution with heat-inactivated (30 min, 56 °C) NMO-IgG serum (1:200; a gift from Vanda A. Lennon, Mayo Clinic, Rochester, MN, USA) for 2 or 10 min at room temperature (RT), followed by fixation in 2% formaldehyde and labeling with anti-human IgG secondary antibodies conjugated to Alexa Fluor 546 (1:600, 20 min, RT; Invitrogen, Carlsbad, CA, USA). Cells were mounted onto glass slides with Slowfade Gold antifade reagent.

Immunocytochemical labeling of intracellular compartments was performed as described previously [19]. Briefly, cells were washed in phosphate-buffered saline (PBS), fixed in 4% formaldehyde solution (15 min, RT; Thermo Fisher Scientific, Waltham, MA, USA) and permeabilized with 0.1% Triton X-100 (10 min, RT). Then, cells were incubated in blocking buffer (3% bovine serum albumin (BSA), 10% goat serum in PBS; 1 h, 37 °C) to prevent background staining, and labeled with primary antibodies (overnight, 4 °C). The primary antibodies used were the rabbit polyclonal antibody to EEA1 (1:500, cat.no.ab2900, Abcam, Cambridge, UK) to label early endosomes, rabbit polyclonal antibody to LAMP1 (1:300, cat.no.ab24170, Abcam, Cambridge, UK) to label late endosomes/lysosomes, and mouse monoclonal antibody to GM130 (1:400, cat.no.610822, BD Biosciences, San Jose, CA, USA) to label the Golgi apparatus. We then incubated the cells with secondary antibodies: goat anti-rabbit or goat anti-mouse IgG secondary antibody conjugated to fluorescent dye Alexa Fluor 546 (1:600, 45 min, 37 °C; cat.no.A11010, Invitrogen, Carlsbad, CA, USA). Samples were mounted onto glass slides using Slowfade Gold antifade reagent (cat.no.A11003, Invitrogen, Carlsbad, CA, USA).

### 2.4. Structured Illumination Microscopy

Z-stack images (500-nm thick) were recorded with an ELYRA PS.1 super-resolution microscope (Zeiss, Jena, Germany), with an oil-immersion Plan-Apochromat DIC objective (63×/NA 1.4) and an EMCCD camera (Andor iXon 885, Andor Technology, Belfast, UK). EGFP and Alexa Fluor 546 were excited with 488 and 561 nm laser beams. The emission fluorescence was filtered with 495–575 nm or 570–650 nm bandpass filters.

### 2.5. Analysis of OAP Density, and Diameter and Area of Intracellular Organelles, OAPs, and MiDs

Image analysis of immunolabeled intracellular organelles, plasmalemmal OAPs, and intracellular punctiform assemblages of AQP4, termed microdomains (MiDs), was performed in Fiji software [35]. MiDs are assemblages of AQP4s where the distance between individual AQP4s is <100 nm (the resolution limit of the structured illumination microscopy (SIM) system [19]). Individual cells were cropped and auto-thresholded (9–3000 px^2^) with the IsoData method. The density of OAPs represents the number of OAPs normalized to the cell surface area.

**Figure 1:**
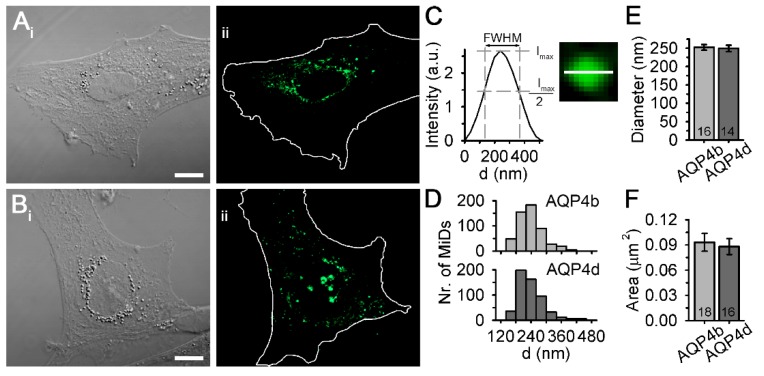
Microdomains (MiDs) of aquaporin (AQP)4b and AQP4d in astrocytes. (**A,B**) DIC and fluorescence micrographs of primary rat astrocytes overexpressing GFP-tagged AQP4b (**A_ii_**) and AQP4d (**B_ii_**). White lines outline cell shapes. Scale bars: **A_i_** (for **A_i_**,**A_ii_**) and **B_i_** (for **B_i_**,**B_ii_**), 10 µm. (**C**) Diameters of MiDs were determined by measuring the full-width at half-maximum (FWHM) of the fluorescence intensity profile along the equatorial line horizontally and vertically (structured illumination microscopy micrograph of AQP4d MiD shows only the horizontal direction; the length of the line is 400 nm). (**D**) Diameter frequency distributions for AQP4b and AQP4d MiDs. The diameters of AQP4b MiDs were in the range of 139–570 nm and the diameters of AQP4d MiDs were in the range of 139–530 nm. (**E**) The average diameter of AQP4b MiDs (252 ± 8 nm) was similar to that of AQP4d MiDs (249 ± 9 nm, *p* = 0.72, Mann–Whitney test). (**F**) The average area of AQP4b MiDs (0.09 ± 0.01 μm^2^) was similar to the average area of AQP4d MiDs (0.09 ± 0.01 μm^2^, *p* = 0.67, Mann–Whitney test). Data represent means ± standard error of the mean from five independent experiments (animals) for AQP4b (*n* = 531 MiDs) and AQP4d (*n* = 548 MiDs) overexpressing astrocytes. The numbers in the bars represent the number of cells analysed.

The diameters of the MiDs were determined by measuring the full-width at half-maximum (FWHM) of the fluorescence intensity profile along the equatorial line (Figure 1C). Areas of OAPs and MiDs were analysed in Fiji software [35]. Individual cells were cropped and auto-thresholded (9–3000 px^2^) with the IsoData method.

### 2.6. Analysis of Colocalization

Colocalization analysis of AQP4b and AQP4d MiDs with selected intracellular compartments was performed in Fiji [35]. Individual cells were cropped and auto-thresholded with the IsoData method. Signals in the range of 9–3000 px^2^ were automatically counted, and split images of MiDs and intracellular compartments were saved superimposed. Colocalized signals were manually counted. The percentage of colocalization was calculated as the percentage of colocalized signals between MiDs and intracellular compartments versus the total fluorescent signal emerging from MiDs.

### 2.7. Cell Volume Measurements

Non-transfected astrocytes and astrocytes transfected with pAQP4b-EGFP or pAQP4d-EGFP were loaded with cytosolic sulphorhodamine 101 dye (SR101; 30 μM; Invitrogen, Carlsbad, CA, USA) for 15 min at 37 °C in 95% air/5% CO_2_. The cells were then stimulated under either isoosmotic (300 mOsm) or hypoosmotic (200 mOsm) conditions and recorded with a laser confocal microscope (LSM 780, Zeiss, Jena, Germany) using an oil-immersion objective (40×/NA 1.3). Transfected cells were confirmed by Ar laser excitation (488 nm) and emitted light was filtered with a bandpass filter at 493–553 nm. SR101 was excited by a diode-pumped solid-state laser (561 nm) and the emission light was filtered with a bandpass filter at 567–649 nm. Time-series images were recorded every 2 s for 120 s (30 s before and 90 s after hypoosmotic stimulation). Changes in the fluorescence intensity of the cytosolic fluorescent dye were used as an indicator of changes in the cell volume [19,26,30]. Recordings were analysed in ZEN 2010 software (Zeiss, Jena, Germany) using the mean region of interest tool, which calculates the average fluorescence intensity, as in our previous study [19]. Detailed analysis and fitting of the data were performed in SigmaPlot 11.0 (SYSTAT, San Jose, CA, USA).

### 2.8. Measurements and Analysis of Vesicle Mobility

The mobility of vesicles in astrocytes transfected with pAQP4b-EGFP and pAQP4d-EGFP was recorded with a confocal microscope (LSM 780, Zeiss, Jena, Germany) using an oil-immersion objective (63×/NA 1.4). EGFP was excited by an Ar laser (488 nm) and the emission light was filtered with a bandpass filter at 493–553 nm. Time-series images of vesicles carrying AQP4b and AQP4d isoforms were recorded at 2-s intervals for 2 min in the following order: (1) in isoosmotic conditions, (2) 2 min after hypoosmotic stimulation (200 mOsm), and (3) 10 min after hypoosmotic stimulation (200 mOsm). Controls were recorded in essentially the same manner, but using isoosmotic solution instead of hypoosmotic solution. Vesicle mobility was analysed in Fiji software [35] using the TrackMate plugin [36].

### 2.9. Statistics

The results are presented as the means ± standard error of the mean. Statistical analysis was performed in SigmaPlot 11.0 (SYSTAT, San Jose, CA, USA). Statistical significance was determined with Student’s *t* test or ANOVA with the Holm–Sidak post hoc test for normally distributed data and the Mann–Whitney test or one-way ANOVA on ranks with the Kruskal–Wallis post hoc test, followed by Dunn’s method for non-normally distributed data. Statistical significance was considered as follows: * *p* < 0.05, ** *p* < 0.01, and *** *p* < 0.001.

## 3. Results

### 3.1. AQP4b and AQP4d Isoforms Are Localized to Organelles and Vesicles in Astrocytes and Are Absent from Plasma Membrane OAPs

We expressed recombinant AQP4b and AQP4d isoforms encoded by fusion proteins AQP4b-GFP (referred to as AQP4b) and AQP4d-GFP (referred to as AQP4d) in primary rat astrocytes. The fluorescence signal of both isoforms was distributed in a punctiform pattern, reminiscent of organelles and vesicles (Figure 1). As previously reported, we termed such punctiform assemblages as MiDs, where, considering the resolution limit of the SIM system, the distance between individual AQP4s is smaller than 100 nm [19]. In SIM micrographs, we assessed the average diameter and the average area of AQP4b and AQP4d MiDs (Figure 1). The diameters of AQP4b and AQP4d MiDs were in the range of 139–570 nm and 139–530 nm Figure 1D), respectively, and their average diameters (*p* = 0.72, Mann–Whitney test; Figure 1E) and average areas (*p* = 0.67, Mann–Whitney test; Figure 1F) were similar.

**Figure 2:**
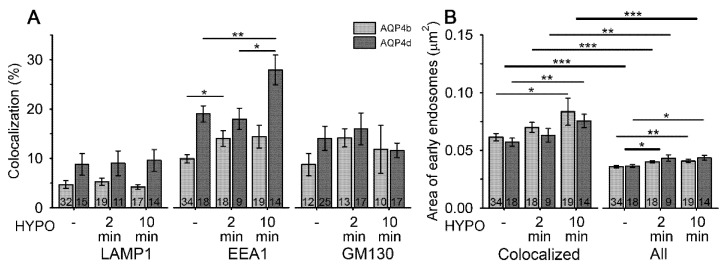
Hypoosmotic conditions trigger the redistribution of AQP4b and AQP4d to early endosomes and increase the area of early endosomes. (**A**) The percentage of colocalization between AQP4b and AQP4d, before and after hypoosmotic exposure, with late endosomes/lysosomes, early endosomes, and the Golgi apparatus, immunolabeled with lysosomal-associated membrane protein 1 (LAMP1), early endosome antigen 1 (EEA1), and Golgi membrane protein GM130, respectively. A significant increase in the colocalization between AQP4b and AQP4d with EEA1 was measured at 2 min (AQP4b) and 10 min (AQP4d) after hypoosmotic stimulation (HYPO) (*p* = 0.03, Kruskal–Wallis test and *p* = 0.006, Holm–Sidak post hoc test, respectively). The percentage of colocalization of AQP4b and AQP4d with late endosomes/lysosomes and the Golgi apparatus remained similar with hypoosmotic stimulation (*p* = 0.35 and *p* = 0.64, respectively, in endosomes/lysosomes; and *p* = 0.07 and *p* = 0.8, respectively, in the Golgi apparatus; Kruskal–Wallis test). (**B**) The average area of early endosomes containing (colocalized) AQP4b and AQP4d isoforms and all endosomes in the cell before and after hypoosmotic exposure in transfected astrocytes. The average area of early endosomes containing AQP4b and AQP4d was significantly larger 10 min after hypoosmotic stimulation (*p* < 0.05, Kruskal–Wallis test and *p* = 0.009, Holm–Sidak post hoc test, respectively). Similarly, the average area of all early endosomes in AQP4b- and AQP4d-transfected cells was significantly larger after 2 and 10 min (AQP4b, *p* = 0.01 and *p* = 0.002, respectively; AQP4d, *p* = 0.01 and *p* = 0.03, respectively, Holm–Sidak post hoc test). Early endosomes containing either AQP4b or AQP4d were twofold larger than all early endosomes (*p* < 0.001, Mann–Whitney test, *p* = 0.007, Student’s t test). Thin lines represent statistical significance between solely AQP4b or AQP4d groups, while thicker lines represent statistical significance observed in both groups containing both isoforms. Data represent means ± standard error of the mean from three (AQP4b) and two (AQP4d) independent experiments (animals). * *p* < 0.05, ** *p* < 0.01, and *** *p* < 0.001. The numbers in the bars represent the number of cells analysed.

The measured dimensions of AQP4b and AQP4d MiDs indicate the possible presence of these two isoforms on different intracellular vesicles. To investigate this issue, we fluorescently counterstained astrocytes overexpressing AQP4b and AQP4d with antibodies against selected organelle markers. We immunolabeled early and late endosomes/lysosomes and the Golgi apparatus with primary antibodies against EEA1, LAMP1, and GM130, respectively (SIM micrographs, Supplementary Figures S1 and S2). Under isoosmotic conditions, both isoforms mostly colocalized with early endosomes (10% ± 1% AQP4b and 19% ± 2% AQP4d; Figure 2), where AQP4d was significantly more abundant than AQP4b (*p* < 0.001, Mann–Whitney test). Both isoforms were similarly colocalized in the Golgi apparatus (9% ± 2% AQP4b and 14% ± 3% AQP4d, *p* = 0.26, Mann–Whitney test). The lowest, and again comparable, colocalization of both isoforms was measured with late endosomes/lysosomes (5% ± 1% AQP4b and 9% ± 2% AQP4d, *p* = 0.22, Mann–Whitney test).

**Figure 3:**
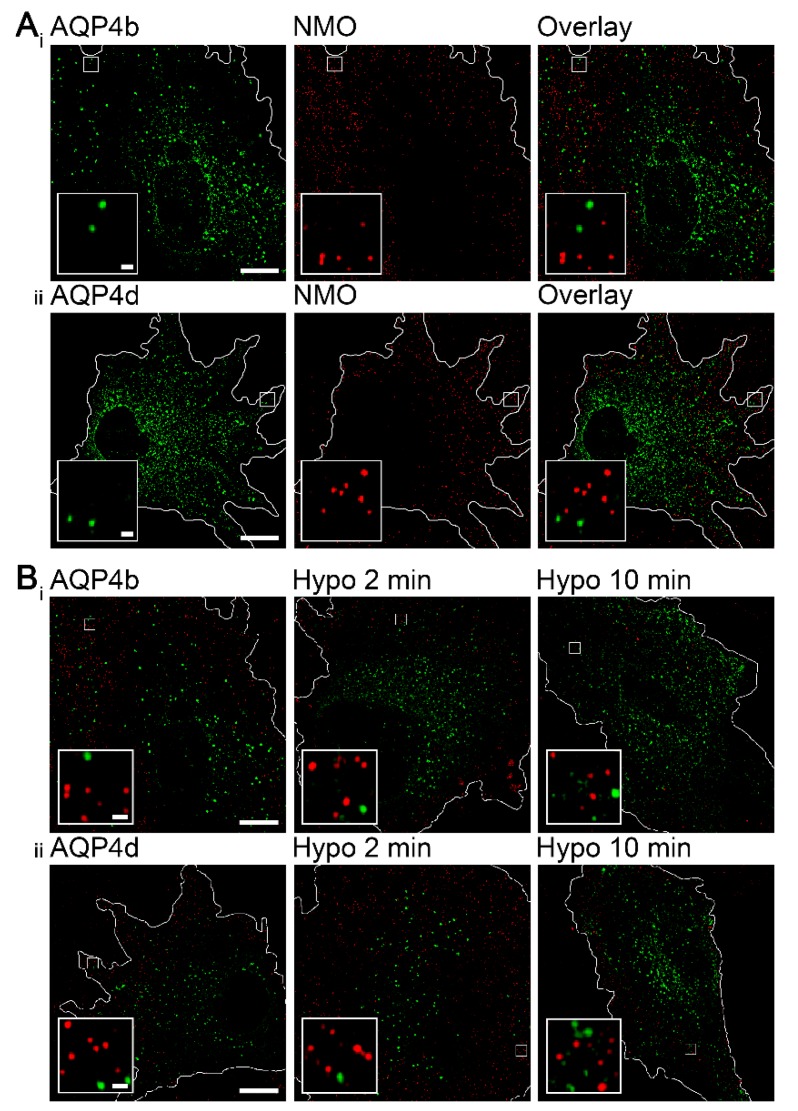
AQP4b and AQP4d are absent from orthogonal arrays of particles (OAPs) in isoosmotic and hypoosmotic conditions. (**A**) Structured illumination microscopy (SIM) micrographs of astrocytes in isoosmotic conditions overexpressing AQP4b or AQP4d. Green spots represent vesicles carrying recombinant AQP4b (**A_i_**) and AQP4d (**A_ii_**), and red spots represent OAPs labeled with NMO-IgG antibodies. Higher magnifications are shown in the insets. Scale bars: 10 µm (inset, 0.5 µm). (**B**) SIM micrographs of astrocytes before and after hypoosmotic stimulation. Green spots represent vesicles carrying recombinant AQP4b (**A_i_**) and AQP4d (**A_ii_**), and red spots represent OAPs labeled with NMO-IgG antibodies. Panels from left to right show astrocytes in isoosmotic conditions and 2 and 10 min after hypoosmotic stimulation. In hypoosmotic conditions, AQP4b and AQP4d isoforms remained absent from OAPs. Higher magnifications are shown in the insets. Scale bars: 10 µm (inset, 0.5 µm).

Considering the inability of AQP4b and AQP4d to transport water, these two isoforms are likely absent from plasmalemmal OAPs, which were shown to consist of water-permeable AQP4 isoforms [19,20,37]. Nevertheless, this fact was investigated. As in previous studies, we used a protocol involving NMO-IgG antibodies to specifically label OAPs [19,21], and found that neither AQP4b nor AQP4d colocalized with OAPs in isoosmotic conditions (Figure 3A,B).

### 3.2. Hypoosmotic Conditions Affect the Localization of AQP4b and AQP4d in Early Endosomes, But Do Not Result in the Redistribution of AQP4b or AQPd to OAPs

Interestingly, hypoosmotic conditions triggered the redistribution of AQP4b and AQP4d, especially to early endosomes (Figure 2). In the case of AQP4b, the localization in early endosomes increased by 41% (*p* = 0.03, Kruskal–Wallis test) 2 min after hypoosmotic challenge and remained at the increased level after 10 min (Figure 2). In contrast, the redistribution of AQP4d was somewhat slower; its presence in early endosomes increased after only 10 min of hypoosmotic stimulation by 47% compared with isoosmotic (-) conditions (*p* = 0.006, Holm–Sidak post hoc test), and by 55% compared with 2 min of hypoosmotic stimulation (*p* = 0.01, Holm–Sidak post hoc test) (Figure 2). Colocalization of AQP4b and AQP4d isoforms with lysosomes and the Golgi apparatus remained virtually unchanged in hypoosmotic versus isoosmotic conditions (*p* = 0.35 and *p* = 0.64, respectively, in lysosomes, Kruskal–Wallis test; and *p* = 0.07 and *p* = 0.8, respectively, in the Golgi apparatus, Kruskal–Wallis test). Hypoosmotic stimulation did not trigger the redistribution of AQP4b or AQP4d into OAPs (Figure 3B).

**Figure 4:**
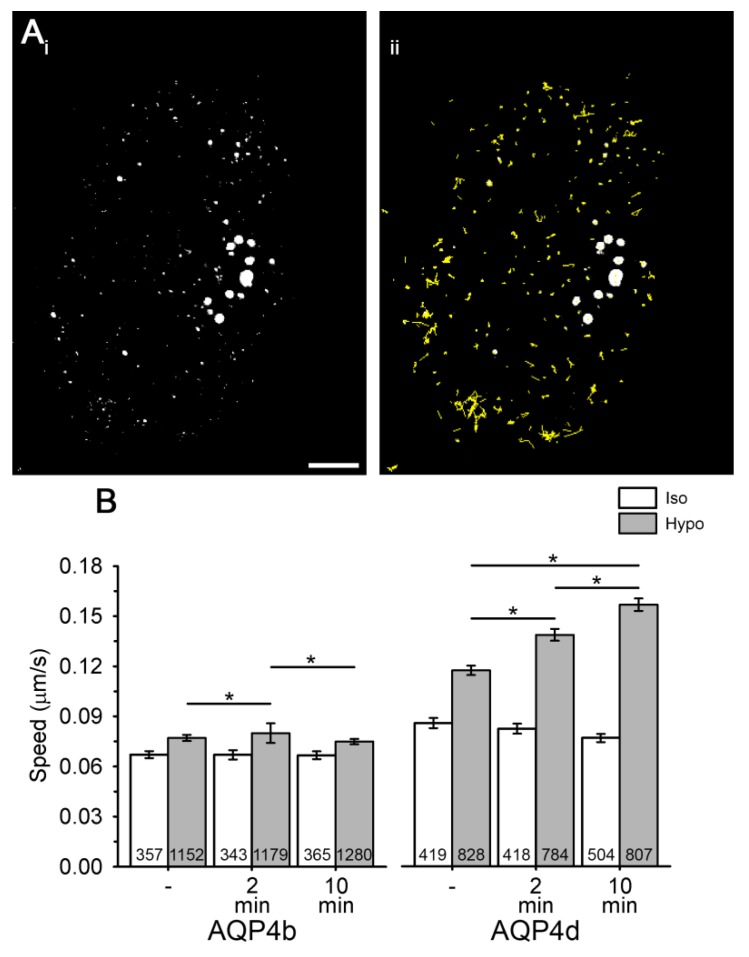
The speed of vesicles carrying recombinant AQP4b and AQP4d increases in hypoosmotic conditions. (**Ai,ii**) Astrocytes overexpressing AQP4d. White spots represent vesicles carrying AQP4d (**i**), and yellow trajectories superimposed over vesicles (**ii**) represent vesicle pathways recorded in a 2-min period in isoosmotic conditions. Scale bar: 10 µm. (**B**) The average speed (µm/s) of AQP4b- and AQP4d-laden vesicles before stimulation (-) and 2 or 10 min after stimulation with isoosmotic (Iso) or hypoosmotic solution (Hypo). Vesicle mobility was recorded at the acquisition rate of 0.5 Hz for 2 min. The numbers in the bars represent the number of astrocytes analysed from three independent experiments, isolated from two animals. Data represent means ± standard error of the mean (* *p* < 0.05, Kruskal–Wallis test, followed by Dunn’s method).

Changes in the localization of AQP4 isoforms in early endosomes may be related to the altered trafficking of vesicles that transport them. To test this hypothesis, we recorded the speed of vesicles containing AQP4b and AQP4d isoforms in isoosmotic and hypoosmotic conditions (Figure 4). Hypoosmotic conditions triggered a smaller transient increase in the speed of AQP4b-laden vesicles after 2 min of hypoosmotic stimulation (by 4%, *p* < 0.05, Kruskal–Wallis test, followed by Dunn’s method; Figure 4B), whereas the speed of AQP4d-laden vesicles showed a steady increase with a longer period of hypoosmotic stimulation (by 18% after 2 min and 34% after 10 min of stimulation, *p* < 0.05, Kruskal–Wallis test, followed by Dunn’s method, Figure 4B). The increase in the colocalization of AQP4b and AQP4d isoforms with early endosomes (Figure 2A) timely coincided with altered vesicle trafficking (Figure 4). Therefore, we tested whether the area of early endosomes increased. The area of early endosomes that were colocalized with either of the two AQP4 isoforms was roughly twofold larger than that of all early endosomes in AQP4b- and AQP4d-transfected cells (*p* < 0.001, Mann–Whitney test and *p* = 0.007, Student’s t test; Figure 2B). Hypoosmotic stimulation induced a significant increase in the average area of early endosomes that colocalized with AQP4b and AQP4d, as well as of all endosomes in cells (Figure 2B).

### 3.3. The Density of OAPs Is Affected by the Overexpression of AQP4b and AQP4d in Isoosmotic Conditions

**Figure 5:**
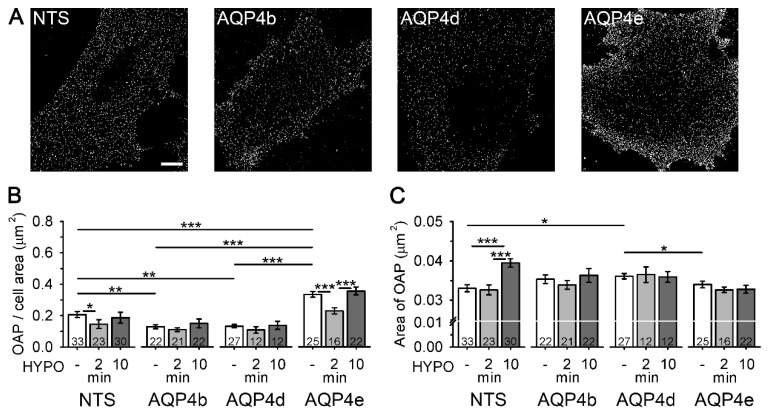
Astrocytes overexpressing AQP4b and AQP4d have a lower density of orthogonal arrays of particles (OAPs). (**A**) SIM micrographs of non-transfected (NTS) astrocytes and astrocytes overexpressing AQP4b, AQP4d, and AQP4e isoforms labeled with NMO-IgG antibodies that tag OAPs (denoted as white dots). Scale bar (for all panels): 10 µm. (**B**) The graph represents the average number of OAPs per cell area (µm^2^) in NTS astrocytes and in astrocytes overexpressing AQP4b, AQP4d, and AQP4e isoforms. In isoosmotic conditions (*-*), astrocytes overexpressing AQP4b and AQP4d showed a significantly lower density of OAPs from NTS astrocytes (*p* = 0.004 and *p* = 0.005, respectively, Mann–Whitney test), and astrocytes overexpressing AQP4e showed a significantly higher density of OAPs from NTS astrocytes (*p* < 0.001, Student’s *t* test). A transient decrease in the density of OAPs in NTS and AQP4e-transfected cells was measured at 2 min of hypoosmotic conditions (*p* = 0.02, Kruskal–Wallis test, *p* < 0.001, Holm–Sidak post hoc test). The density of OAPs in cells overexpressing AQP4b or AQP4d was similar in isoosmotic and hypoosmotic conditions (*p* = 0.64 and *p* = 0.36, Kruskal–Wallis test). (**C**) The graph represents the average area of OAPs in isoosmotic (-) and hypoosmotic (HYPO) conditions. In isoosmotic conditions, astrocytes overexpressing AQP4d had larger OAPs than NTS cells (*p* = 0.01, Student’s *t* test) and cells overexpressing AQP4e (*p* = 0.03, Mann–Whitney test). In hypoosmotic conditions (10 min HYPO), an increase in the OAP area was measured in NTS cells (*p* < 0.001, Holm–Sidak post hoc test). Data represent means ± standard error of the mean from independent experiments equal to the number of animals: 4 (NTS), 3 (AQP4b and AQP4d), and 2 (AQP4e) animals. * *p* < 0.05, ** *p* < 0.01, and *** *p* < 0.001. The numbers in the bars represent the number of cells analysed.

The results showing that AQP4b and AQP4d are absent from OAPs have ruled out the possibility that these two isoforms are directly involved in water transport through the plasmalemma of astrocytes. However, in hypoosmotic conditions, we detected redistribution of the respective isoforms in endosomes. Considering these changes in the intracellular localization of AQP4b and AQP4d, we tested whether these two isoforms affected the density of OAPs (and hence the water transport) indirectly. Therefore, we measured the density of OAPs in the plasma membrane of astrocytes overexpressing AQP4b and AQP4d. Non-transfected (NTS) astrocytes and astrocytes overexpressing isoforms AQP4b, AQP4d, and AQP4e were labeled with NMO-IgG antibodies to visualize OAPs (Figure 5A). The density of OAPs, expressed as the number of OAPs normalized to the cell surface area (i.e., two-dimensional projections of the cell), was then determined from SIM micrographs (Figure 5B). A comparison of the density of OAPs between NTS cells and cells overexpressing different AQP4 isoforms revealed that, in isoosmotic conditions, cells overexpressing AQP4b and AQP4d exhibited a reduced (by 37%) density of OAPs compared with NTS cells (*p* = 0.004 and *p* = 0.005, respectively, Mann–Whitney test). In contrast, the overexpression of AQP4e increased the density of OAPs by 61% in comparison with NTS cells (*p* < 0.001, Student’s *t* test) and by 154% compared with cells overexpressing AQP4b and AQP4d (*p* < 0.001, Mann–Whitney test, Figure 5B).

Hypoosmotic stimulation of NTS astrocytes triggered a transient decrease in the density of OAPs (*p* = 0.02, Kruskal–Wallis test), whereas in cells overexpressing AQP4b and AQP4d, no significant change in the density of OAPs was detected compared with isoosmotic conditions (*p* = 0.64 and *p* = 0.36, respectively, Kruskal–Wallis test; Figure 5B). On the other hand, in cells transfected with functional water channel AQP4e, a 32% transient decrease in the density of OAPs was detected (even more prominent than 30% in NTS cells), compared with isoosmotic conditions (*p* < 0.001, Holm–Sidak post hoc test; Figure 5B), followed by an increase after 10 min of hypoosmotic stimulation (55%), which was again more prominent than in NTS cells (28%).

Next, we tested whether the overexpression of particular AQP4 isoforms can affect the size of individual OAPs (Figure 5C). In isoosmotic conditions, the average area of OAPs was 9% larger in astrocytes overexpressing AQP4d compared with NTS cells (*p* = 0.01, Student’s *t* test), and 6% larger compared with cells overexpressing AQP4e (*p* = 0.03, Mann–Whitney test). The average OAP area in astrocytes overexpressing AQP4b was also larger than in NTS cells (7%), but the difference was not significant (*p* = 0.12, Student’s *t* test; Figure 5C). Hypoosmotic conditions only affected the size of OAPs in NTS cells (Figure 5C). The average OAP area was 20% larger after 10 min of hypoosmotic stimulation versus isoosmotic conditions (*p* < 0.001, Holm–Sidak post hoc test). However, when we overexpressed AQP4b, AQP4d, or AQP4e, the area of OAPs appeared unaffected by hypoosmotic stimulation (*p* = 0.69, *p* = 0.86 and *p* = 0.36, respectively, Kruskal–Wallis test; Figure 5C).

### 3.4. Overexpression of AQP4d Affects Cell Volume Changes in Hypoosmotic Conditions

The significant redistribution of AQP4b and AQP4d isoforms in early endosomes in hypoosmotic conditions (Figure 2) and the impact of AQP4b and AQP4d overexpression on the density of OAPs in isoosmotic conditions (Figure 5B) indicate that these two isoforms may indirectly affect volume changes in astrocytes.

**Figure 6:**
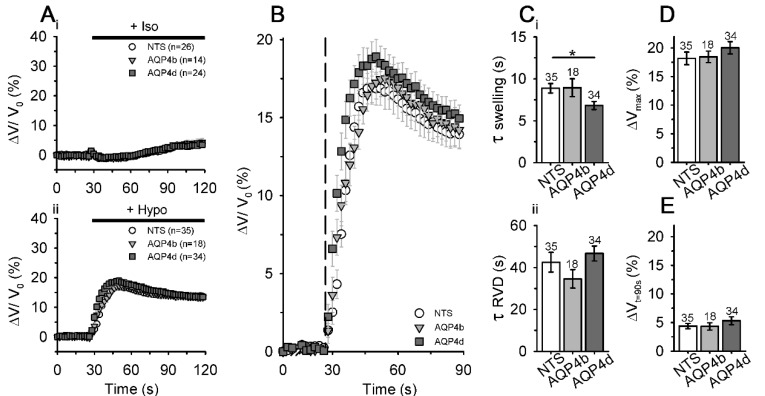
Astrocytes overexpressing AQP4d swell faster in hypoosmotic conditions. (**A**) The average relative volume changes, obtained by measuring the average fluorescence intensity of sulphorhodamine 101 (SR101) in non-transfected (NTS) astrocytes, and in astrocytes overexpressing AQP4b or AQP4d in isoosmotic (Iso; **A_i_**) and hypoosmotic (Hypo; **A_ii_**) conditions. (**B**) The expanded section of the graph shown in **A_ii_** represents the swelling phase (Δ*V*/*V*_0_) in astrocytes after hypoosmotic stimulation (the addition of hypoosmotic stimulation is represented by the dashed line). (**C_i_**) The swelling phase (τ swelling) was faster in cells overexpressing AQP4d than in NTS cells (*p* < 0.05, Kruskal–Wallis test, followed by Dunn’s method), whereas in cells overexpressing AQP4b, the swelling phase was similar to that in NTS cells (*p* > 0.05, Kruskal–Wallis test, followed by Dunn’s method). (**C_ii_**) Time constants of the regulatory volume decrease (RVD) phase were similar for NTS cells and cells overexpressing AQP4b or AQP4d (*p* = 0.17, Kruskal–Wallis test). (**D**) Maximal amplitude of the volume increase (Δ*V*_max_) and (**E**) recovery of the cell volume at 90 s (Δ*V_t_*_=90s_) in the RVD phase were similar for NTS cells and cells overexpressing AQP4b or AQP4d (*p* = 0.44 and *p* = 0.77, respectively, Kruskal–Wallis test). Data represent means ± standard error of the mean from independent experiments equal to the number of animals: 6 (NTS), 3 (AQP4b), and 4 (AQP4d). The numbers above the bars represent the number of cells analysed.

To test the hypothesis that AQP4b and AQP4d indirectly affect fluctuations in the cell volume in hypoosmotic conditions, we labeled NTS cells and cells overexpressing AQP4b and AQP4d with sulphorhodamine 101 dye to measure changes in the cell volume after stimulation with hypoosmotic solution, as described previously [19]. We would like to stress that the method was calibrated in a previous study by the AFM technique [19], which has a superb spatial resolution, yet it lacks temporal resolution, compared with fluorescence measurements. Furthermore, measurements were performed at room temperature, at which point AQP4’s impact on water transport is amplified, compared to physiological 37 ℃. Hence, the absolute volume changes determined with our method may be underestimated [38]. NTS cells and cells overexpressing AQP4b and AQP4d exposed to isoosmotic conditions showed little fluctuations in cell volume throughout the recording time (120 s; Figure 6A_i_).

On the other hand, cells exposed to hypoosmotic solution swelled rapidly and then, to balance sudden changes in the cellular volume, the cell volume quickly started to decrease initially and then slowly approached a new steady state (Figure 6A_ii_,B). The time constant of swelling (τ swelling) and the time constant of RVD (τ RVD) were similar for NTS cells (τ swelling 8.9 ± 0.6 s, τ RVD 42.5 ± 4.7 s) and cells overexpressing AQP4b (τ swelling 8.9 ± 1.1 s, τ RVD 34.6 ± 4.4 s, *p* > 0.05, Kruskal–Wallis test, followed by Dunn’s method; Figure 6C). Upon the application of hypoosmotic solution, cells overexpressing AQP4d swelled significantly faster (τ swelling 6.8 ± 0.5 s, *p* < 0.05, Kruskal–Wallis test, followed by Dunn’s method) than NTS cells, but the rate for the RVD phase (τ RVD 46.7 ± 3.6 s) remained similar to that of NTS cells (*p* = 0.17, Kruskal–Wallis test; Figure 6C). NTS cells and cells overexpressing AQP4b or AQP4d also showed a similar maximal amplitude of the volume increase and recovery of the cell volume in the RVD phase (*p* = 0.44 and 0.77, respectively, Kruskal–Wallis test; Figure 6D,E).

In summary, the overexpression of AQP4b did not affect cell volume changes, whereas the overexpression of AQP4d significantly affected the speed of cell swelling, but not the RVD and maximal cell volume increase, in hypoosmotic conditions.

## 4. Discussion

In the present study, we show that isoforms AQP4b and AQP4d reside in intracellular structures and remain there in hypoosmotic conditions. Nonetheless, hypoosmotic conditions triggered a dynamic relocation of both isoforms to early endosomes, where a particularly conspicuous increase of AQP4d was observed, as well as an increase in the average area of early endosomes. Hypotonicity-induced relocation of the two AQP4 isoforms was most likely mediated by the altered trafficking speed of vesicles carrying recombinant AQP4b and especially AQP4d isoforms. We also confirmed that the expression of the AQP4d isoform results in functional changes related to astrocyte swelling. The overexpression of both isoforms affected the density of OAPs in isoosmotic conditions, and the overexpression of AQP4d affected the speed of cell swelling in hypoosmotic conditions.

Our results, obtained in primary rat astrocytes by using SIM, show that isoforms AQP4b and AQP4d are intracellular. The size of AQP4b and AQP4d MiDs in cells overexpressing AQP4b and AQP4d isoforms was larger than in previous measurements of MiDs in NTS astrocytes and in astrocytes overexpressing predominant plasmalemmal AQP4e [19]. Nevertheless, the size distribution of MiDs containing AQP4b and AQP4d is in good agreement with the diameter of various vesicles reported for astrocytes [39,40]. The localization of both AQP4 isoforms was indeed confirmed in late endosomes/lysosomes, early endosomal compartments, and the Golgi apparatus. Previous reports have already associated AQP4b and AQP4d with the Golgi apparatus in the HeLa cell line [12,15], and AQP4d was also found to be colocalized with the Golgi apparatus and late endosomes/lysosomes in primary astrocytes [12]. The localization of either isoform with other organelles was not tested in any other cell type. Although our previous study indicated the possible presence of AQP4d at the plasma membrane, which was labeled with lipophilic dye, we report here the absence of AQP4d, as well as AQP4b, from plasma membrane OAPs. The discrepancies regarding the distribution of AQP4d in the plasma membrane in these two reports probably arise from different labeling methods of the plasma membrane areas and from the use of microscopies with different spatial resolutions. First, labeling with NMO-IgG antibodies is essentially different from general staining of the plasma membrane, because dual-labeled domains are undoubtedly located in the plasmalemma [21]. On the other hand, AQP4d present in early endosomes or some other organelle lying near the plasma membrane (i.e., closer than the resolution of the confocal microscope; ~200 nm laterally or even 500 nm axially) stained with lipophilic dye may appear plasmalemmal when inspected with confocal microscopy [12]. In hypoosmotic conditions, a significant redistribution of AQP4b, and especially the AQP4d isoform, into early endosomes was detected. In the same time span after hypoosmotic stimulation, significant changes in the vesicle speed of vesicles carrying AQP4b and AQP4d were measured. An increase in the speed of AQP4b-laden vesicles was transient in nature, but AQP4d-laden vesicles showed a remarkably steady increase in the average speed throughout the experiment. Altered parameters of vesicle trafficking in hypoosmotic conditions in rat astrocytes were also reported for vesicles carrying the AQP4e isoform [12]. In contrast to the mobility of intracellular AQP4 isoforms, the mobility of AQP4e, a plasmalemmal AQP4, was transiently reduced following the hypotonic treatment [12]. This indicates that hypotonicity differentially affects the mobility of both pools of vesicles—vesicles containing intracellular AQP4 isoforms move faster, while vesicles containing plasmalemmal AQP4 isoforms are transiently slower when cells are exposed to hypotonicity. A redistribution of AQP4b or AQP4d was not detected in the Golgi apparatus and in late endosomes/lysosomes. These results, together with the fact that of all the inspected organelles, the highest percentage of both isoforms was observed in early endosomes, indicate that early endosomes play an important part in the positioning of intracellular isoforms AQP4b and AQP4d under different osmotic conditions.

The measurements of the relocation of AQP4b and AQP4d in early endosomes in hypoosmotic conditions, which correlated well with the increased speed of vesicles laden with AQP4b or AQP4d isoforms, point to the importance of the redistribution of AQP4 isoforms through vesicle delivery. The hypoosmotically-triggered redistribution (increased colocalization) of intracellular AQP4 isoforms to early endosomes coincided with an increase in the average area of early endosomes. The pool of early endosomes was predicted to be important for the redistribution of AQP4 between the intracellular space and the plasma membrane in primary rat astrocytes and in vivo, which could potentially influence the water transport across cell membranes [41,42,43]. This is not a unique characteristic of AQP4, because early endosomes were considered to be important for the redistribution of another aquaporin—AQP2 [44,45]. AQP2 is retained in the cytoplasm of collecting duct cells in the kidney, and it is transiently translocated to the plasma membrane after antidiuretic hormone (vasopressin) stimulation, presumably from the pool of early endosomes [44].

It appears that changes in the trafficking of AQP4b- and AQP4d-laden vesicles in hypoosmotic conditions predominantly affected their localization in early endosomes, because these two isoforms did not distribute into OAPs. Although AQP4b and AQPd isoforms were absent from OAPs, they still managed to affect the density of OAPs in astrocytes in isoosmotic conditions and the overexpression of both isoforms resulted in an increase of the average area of OAPs per se. This finding may indicate that these two intracellular isoforms may indirectly affect the formation of OAPs, apparently through changes taking place in early endosomes. However, the lower density and larger size of OAPs could also be the consequence of an OAP merger as a consequence of membrane diffusion; however, this is unlikely, since AQP4 M23, which forms OAPs, is nearly immobile [23]. Similar to our findings, another intracellular isoform of the AQP4 channel was found to indirectly affect the density of OAPs. The AQP4-Δ4 isoform in skeletal muscle, similar to AQP4b and AQP4d, lacks the structural properties required to be a fully functional water channel [18]. The expression of AQP4-Δ4 in the HeLa cell line massively reduced the abundance of the overall AQP4 signal and OAPs in the plasma membrane [18]. In a human gastric adenocarcinoma cell line transfected with rat M1 AQP4, the endosomal route was found to be linked to AQP4 redistribution, because AQP4 was identified in late endosomes, from where it is assumed to recycle back to the plasma membrane after histamine washout [46].

Motivated by the changes observed in the redistribution of AQP4b and AQP4d in early endosomes in hypoosmotic conditions and by the (indirect) effect they had on the density and size of OAPs, we tested whether AQP4b and AQP4d also affect volume changes in astrocytes. Although the overexpression of AQP4b failed to exert any changes in the cell swelling and consequent RVD after hypoosmotic stimulation, the overexpression of AQP4d triggered significantly faster cell swelling, but the kinetics of the RVD phase, maximal changes in the cell volume, and the recovery phase remained similar to NTS cells. Compared with the effect of AQP4e, the effect of AQP4d on cell swelling was somewhat less extensive [19], which is to be expected, because AQP4e is a fully functional water channel [15]. Observed changes in the swelling of cells overexpressing AQP4d, as well as the absence of effects in the volume recovery, coincided with the increase in the average area of OAPs (which was observed in cells overexpressing AQP4d, but was already absent 2 minutes after hypotonic stimulation). At the same time, we have also noticed that the density of OAPs in cells overexpressing AQP4b and AQP4d is lower. Moreover, considering that the permeability of individual AQP4 isoforms in OAPs differs [47], alterations in the density and area of OAPs should be interpreted with caution.

Although AQP4b and AQP4d expression in *Xenopus* oocytes failed to enhance water permeability through their plasma membrane [15], it appears that they may indirectly affect astrocyte volume changes, possibly through affecting the presence and/or assembly of other AQP4 isoforms in the plasma membrane through sorting in early endosomes. Endosomes are in general increasingly recognized as organelles importantly implicated in the spatial and temporal compartmentalization of signal transduction contributing to signaling specificity and regulation [48,49]. Early endosomes at “the crossroad” in the paths of aquaporins are emerging as important players in the sorting of different isoforms and types of aquaporin channels, and therefore in the regulation of water transport through the plasma membrane. The underlying mechanisms responsible for the presence of AQP4 at the plasmalemma of astrocytes are complex and linked to the dynamics of the internalization and delivery of AQP4 channels to the cell surface. Along with the per se AQP4 presence in the plasmalemma, the ratio of the AQP4 isoforms in OAPs also affects water transport [50,51]; in addition, our experiments have shown that the intracellular AQP4 isoforms are also important in regulating the abundance and size of OAPs in astrocytes. In pathophysiologic conditions, i.e., in the ischemic brain edema, AQP4 is shown to internalize and colocalize with early ensodome marker EEA1, as was demonstrated in rat brain sections [42]. Early endosomes are the first endocytotic compartment where endocytosed vesicles (also the ones laden with OAP-forming AQP4 isoforms) unite with vesicles from the Golgi complex (among others, the ones containing intracellular AQP4 isoforms). The fusion of vesicles mediates the merging of all incoming vesicles with early endosomes, while fission redirects outgoing vesicles that bud from early endosomes (to the recycling or degradation pathway) and as such, early endosomes represent major sorting platforms [52]. The molecular mechanisms behind the sorting and budding of vesicles from early endosomes are challenging to study, because of experimental limitations related to the reconstitution of sorting from endosomes in vitro. Nonetheless, our results imply that endosomes containing AQP4b or AQP4d have the recycling pathway either fully blocked, or a mechanism exists that prevents the budding of recycling vesicles with these two isoforms (since AQP4b and AQP4d are not present in the plasmalemma). Such a blockade of one of the outgoing trafficking pathways would likely lead to an increase in the size of these organelles, which we have indeed observed. In addition, if endocytic vesicles containing any of the OAP-forming isoforms would fuse with early endosome-containing intracellular AQP4 isoforms, then recycling back to the plasmalemma would be impaired as well. In agreement, we have observed lower densities of OAPs in astrocytes overexpressing AQP4b or AQP4d. While this topic is currently still not fully resolved, it appears that higher order complexes consist of at least three isoforms: AQP4c (M23), and also invariably AQP4a (M1) and/or AQP4 e (Mz) [19,53,54]. The high curvature and small size of endocytic vesicles likely preclude endocytosis of the whole OAP [25]; however, the binding of an antibody that recognized M1 and M23 outside OAPs enhanced the endocytosis of both, followed by degradation in astrocytes, in contrast to an antibody that almost exclusively recognized M23 [55]. This process is especially relevant to certain pathologies, including neuromyelitis optica, where antibodies target AQP4 and can result in the internalization of plasmalemmal isoforms of AQP4, which are then passed on to early endosomes [56]. Further studies are needed to elucidate the role of early endosomes and the role of different AQP4 isoforms in the regulation of water balance in astrocytes.

## Supplementary Materials

The following are available online at https://www.mdpi.com/2073-4409/9/3/735/s1, Figure S1: Colocalization of AQP4b with selected intracellular compartments in astrocytes. Figure S2: Colocalization of AQP4d with selected intracellular compartments in astrocytes.

## References

[1] Jung J.S., Bhat R.V., Preston G.M., Guggino W.B., Baraban J.M., Agre P. (1994). Molecular characterization of an aquaporin cDNA from brain: Candidate osmoreceptor and regulator of water balance. Proc. Natl. Acad. Sci. USA.

[2] King L.S., Kozono D., Agre P. (2004). From structure to disease: The evolving tale of aquaporin biology. Nat. Rev. Mol. Cell Biol..

[3] Tani K., Mitsuma T., Hiroaki Y., Kamegawa A., Nishikawa K., Tanimura Y., Fujiyoshi Y. (2009). Mechanism of aquaporin-4′s fast and highly selective water conduction and proton exclusion. J. Mol. Biol..

[4] Nagelhus E.A., Ottersen O.P. (2013). Physiological roles of aquaporin-4 in brain. Physiol. Rev..

[5] Potokar M., Jorgačevski J., Zorec R. (2016). Astrocyte Aquaporin Dynamics in Health and Disease. Int. J. Mol. Sci..

[6] Badaut J., Fukuda A.M., Jullienne A., Petry K.G. (2014). Aquaporin and brain diseases. Biochim. Biophys. Acta.

[7] Frigeri A., Gropper M.A., Umenishi F., Kawashima M., Brown D., Verkman A.S. (1995). Localization of MIWC and GLIP water channel homologs in neuromuscular, epithelial and glandular tissues. J. Cell Sci..

[8] Nielsen S., Nagelhus E.A., Amiry-Moghaddam M., Bourque C., Agre P., Ottersen O.P. (1997). Specialized membrane domains for water transport in glial cells: High-resolution immunogold cytochemistry of aquaporin-4 in rat brain. J. Neurosci..

[9] Rash J.E., Yasumura T., Hudson C.S., Agre P., Nielsen S. (1998). Direct immunogold labeling of aquaporin-4 in square arrays of astrocyte and ependymocyte plasma membranes in rat brain and spinal cord. Proc. Natl. Acad. Sci. USA.

[10] Nagelhus E.A., Mathiisen T.M., Ottersen O.P. (2004). Aquaporin-4 in the central nervous system: Cellular and subcellular distribution and coexpression with KIR4.1. Neuroscience.

[11] Nicchia G.P., Rossi A., Mola M.G., Procino G., Frigeri A., Svelto M. (2008). Actin cytoskeleton remodeling governs aquaporin-4 localization in astrocytes. Glia.

[12] Potokar M., Stenovec M., Jorgacevski J., Holen T., Kreft M., Ottersen O.P., Zorec R. (2013). Regulation of AQP4 surface expression via vesicle mobility in astrocytes. Glia.

[13] Hasegawa H., Ma T., Skach W., Matthay M.A., Verkman A.S. (1994). Molecular cloning of a mercurial-insensitive water channel expressed in selected water-transporting tissues. J. Biol. Chem..

[14] Neely J.D., Christensen B.M., Nielsen S., Agre P. (1999). Heterotetrameric composition of aquaporin-4 water channels. Biochemistry.

[15] Moe S.E., Sorbo J.G., Sogaard R., Zeuthen T., Petter Ottersen O., Holen T. (2008). New isoforms of rat Aquaporin-4. Genomics.

[16] Lu M., Lee M.D., Smith B.L., Jung J.S., Agre P., Verdijk M.A., Merkx G., Rijss J.P., Deen P.M. (1996). The human AQP4 gene: Definition of the locus encoding two water channel polypeptides in brain. Proc. Natl. Acad. Sci. USA.

[17] Yang B., Ma T., Verkman A.S. (1995). cDNA cloning, gene organization, and chromosomal localization of a human mercurial insensitive water channel. Evidence for distinct transcriptional units. J. Biol. Chem..

[18] De Bellis M., Pisani F., Mola M.G., Basco D., Catalano F., Nicchia G.P., Svelto M., Frigeri A. (2014). A novel human aquaporin-4 splice variant exhibits a dominant-negative activity: A new mechanism to regulate water permeability. Mol. Biol. Cell.

[19] Lisjak M., Potokar M., Rituper B., Jorgacevski J., Zorec R. (2017). AQP4e-Based Orthogonal Arrays Regulate Rapid Cell Volume Changes in Astrocytes. J. Neurosci..

[20] Nicchia G.P., Rossi A., Mola M.G., Pisani F., Stigliano C., Basco D., Mastrototaro M., Svelto M., Frigeri A. (2010). Higher order structure of aquaporin-4. Neuroscience.

[21] Nicchia G.P., Mastrototaro M., Rossi A., Pisani F., Tortorella C., Ruggieri M., Lia A., Trojano M., Frigeri A., Svelto M. (2009). Aquaporin-4 orthogonal arrays of particles are the target for neuromyelitis optica autoantibodies. Glia.

[22] Furman C.S., Gorelick-Feldman D.A., Davidson K.G., Yasumura T., Neely J.D., Agre P., Rash J.E. (2003). Aquaporin-4 square array assembly: Opposing actions of M1 and M23 isoforms. Proc. Natl. Acad. Sci. USA.

[23] Crane J.M., Van Hoek A.N., Skach W.R., Verkman A.S. (2008). Aquaporin-4 dynamics in orthogonal arrays in live cells visualized by quantum dot single particle tracking. Mol. Biol. Cell.

[24] Crane J.M., Bennett J.L., Verkman A.S. (2009). Live cell analysis of aquaporin-4 m1/m23 interactions and regulated orthogonal array assembly in glial cells. J. Biol. Chem..

[25] Rossi A., Baumgart F., van Hoek A.N., Verkman A.S. (2012). Post-Golgi supramolecular assembly of aquaporin-4 in orthogonal arrays. Traffic.

[26] Mola M.G., Sparaneo A., Gargano C.D., Spray D.C., Svelto M., Frigeri A., Scemes E., Nicchia G.P. (2016). The speed of swelling kinetics modulates cell volume regulation and calcium signaling in astrocytes: A different point of view on the role of aquaporins. Glia.

[27] Risher W.C., Andrew R.D., Kirov S.A. (2009). Real-time passive volume responses of astrocytes to acute osmotic and ischemic stress in cortical slices and in vivo revealed by two-photon microscopy. Glia.

[28] Pasantes-Morales H., Lezama R.A., Ramos-Mandujano G., Tuz K.L. (2006). Mechanisms of cell volume regulation in hypo-osmolality. Am. J. Med..

[29] Olson J.E., Sankar R., Holtzman D., James A., Fleischhacker D. (1986). Energy-dependent volume regulation in primary cultured cerebral astrocytes. J. Cell Physiol..

[30] Benfenati V., Caprini M., Dovizio M., Mylonakou M.N., Ferroni S., Ottersen O.P., Amiry-Moghaddam M. (2011). An aquaporin-4/transient receptor potential vanilloid 4 (AQP4/TRPV4) complex is essential for cell-volume control in astrocytes. Proc. Natl. Acad. Sci. USA.

[31] Lennon V.A., Kryzer T.J., Pittock S.J., Verkman A.S., Hinson S.R. (2005). IgG marker of optic-spinal multiple sclerosis binds to the aquaporin-4 water channel. J. Exp. Med..

[32] Lennon V.A., Wingerchuk D.M., Kryzer T.J., Pittock S.J., Lucchinetti C.F., Fujihara K., Nakashima I., Weinshenker B.G. (2004). A serum autoantibody marker of neuromyelitis optica: Distinction from multiple sclerosis. Lancet.

[33] Ratelade J., Bennett J.L., Verkman A.S. (2011). Evidence against cellular internalization in vivo of NMO-IgG, aquaporin-4, and excitatory amino acid transporter 2 in neuromyelitis optica. J. Biol. Chem..

[34] Bradl M., Lassmann H. (2014). Experimental models of neuromyelitis optica. Brain Pathol..

[35] Schindelin J., Arganda-Carreras I., Frise E., Kaynig V., Longair M., Pietzsch T., Preibisch S., Rueden C., Saalfeld S., Schmid B. (2012). Fiji: An open-source platform for biological-image analysis. Nat. Methods.

[36] Tinevez J.Y., Perry N., Schindelin J., Hoopes G.M., Reynolds G.D., Laplantine E., Bednarek S.Y., Shorte S.L., Eliceiri K.W. (2017). TrackMate: An open and extensible platform for single-particle tracking. Methods.

[37] Rossi A., Moritz T.J., Ratelade J., Verkman A.S. (2012). Super-resolution imaging of aquaporin-4 orthogonal arrays of particles in cell membranes. J. Cell Sci..

[38] Solenov E., Watanabe H., Manley G.T., Verkman A.S. (2004). Sevenfold-reduced osmotic water permeability in primary astrocyte cultures from AQP-4-deficient mice, measured by a fluorescence quenching method. Am. J. Physiol. Cell Physiol..

[39] Gucek A., Jorgacevski J., Singh P., Geisler C., Lisjak M., Vardjan N., Kreft M., Egner A., Zorec R. (2016). Dominant negative SNARE peptides stabilize the fusion pore in a narrow, release-unproductive state. Cell Mol. Life Sci..

[40] Verkhratsky A., Matteoli M., Parpura V., Mothet J.P., Zorec R. (2016). Astrocytes as secretory cells of the central nervous system: Idiosyncrasies of vesicular secretion. EMBO J..

[41] Tham D.K., Joshi B., Moukhles H. (2016). Aquaporin-4 Cell-Surface Expression and Turnover Are Regulated by Dystroglycan, Dynamin, and the Extracellular Matrix in Astrocytes. PLoS ONE.

[42] Huang J., Sun S.Q., Lu W.T., Xu J., Gan S.W., Chen Z., Qiu G.P., Huang S.Q., Zhuo F., Liu Q. (2013). The internalization and lysosomal degradation of brain AQP4 after ischemic injury. Brain Res..

[43] Xu J., Qiu G.P., Huang J., Zhang B., Sun S.Q., Gan S.W., Lu W.T., Wang K.J., Huang S.Q., Zhu S.J. (2015). Internalization of aquaporin-4 after collagenase-induced intracerebral hemorrhage. Anat. Rec. (Hoboken).

[44] Tajika Y., Matsuzaki T., Suzuki T., Aoki T., Hagiwara H., Tanaka S., Kominami E., Takata K. (2002). Immunohistochemical characterization of the intracellular pool of water channel aquaporin-2 in the rat kidney. Anat. Sci. Int..

[45] Moeller H.B., Knepper M.A., Fenton R.A. (2009). Serine 269 phosphorylated aquaporin-2 is targeted to the apical membrane of collecting duct principal cells. Kidney Int..

[46] Carmosino M., Procino G., Tamma G., Mannucci R., Svelto M., Valenti G. (2007). Trafficking and phosphorylation dynamics of AQP4 in histamine-treated human gastric cells. Biol. Cell.

[47] Fenton R.A., Moeller H.B., Zelenina M., Snaebjornsson M.T., Holen T., MacAulay N. (2010). Differential water permeability and regulation of three aquaporin 4 isoforms. Cell Mol. Life Sci..

[48] Miaczynska M., Pelkmans L., Zerial M. (2004). Not just a sink: Endosomes in control of signal transduction. Curr Opin Cell Biol..

[49] Villaseñor R., Kalaidzidis Y., Zerial M. (2016). Signal processing by the endosomal system. Curr. Opin. Cell Biol..

[50] Tajima M., Crane J.M., Verkman A.S. (2010). Aquaporin-4 (AQP4) associations and array dynamics probed by photobleaching and single-molecule analysis of green fluorescent protein-AQP4 chimeras. J. Biol. Chem..

[51] Jin B.J., Rossi A., Verkman A.S. (2011). Model of aquaporin-4 supramolecular assembly in orthogonal arrays based on heterotetrameric association of M1-M23 isoforms. Biophys. J..

[52] Barysch S.V., Aggarwal S., Jahn R., Rizzoli S.O. (2009). Sorting in early endosomes reveals connections to docking- and fusion-associated factors. Proc. Natl. Acad. Sci. USA.

[53] Sorbo J.G., Moe S.E., Ottersen O.P., Holen T. (2008). The molecular composition of square arrays. Biochemistry.

[54] Strand L., Moe S.E., Solbu T.T., Vaadal M., Holen T. (2009). Roles of aquaporin-4 isoforms and amino acids in square array assembly. Biochemistry.

[55] Huang P., Takai Y., Kusano-Arai O., Ramadhanti J., Iwanari H., Miyauchi T., Sakihama T., Han J.Y., Aoki M., Hamakubo T. (2016). The binding property of a monoclonal antibody against the extracellular domains of aquaporin-4 directs aquaporin-4 toward endocytosis. Biochem. Biophys. Rep..

[56] Hinson S.R., Romero M.F., Popescu B.F., Lucchinetti C.F., Fryer J.P., Wolburg H., Fallier-Becker P., Noell S., Lennon V.A. (2012). Molecular outcomes of neuromyelitis optica (NMO)-IgG binding to aquaporin-4 in astrocytes. Proc. Natl. Acad. Sci. USA.

