# Supplementary material for: Indirect Role of AQP4b and AQP4d Isoforms in Dynamics of Astrocyte Volume and Orthogonal Arrays of Particles
Source: Cells. 2020 Mar 17;9(3):735. doi: 10.3390/cells9030735 (PMC7140617; doi:10.3390/cells9030735)
Supplement: Supplementary file 1 [file cells-09-00735-s001.pdf]

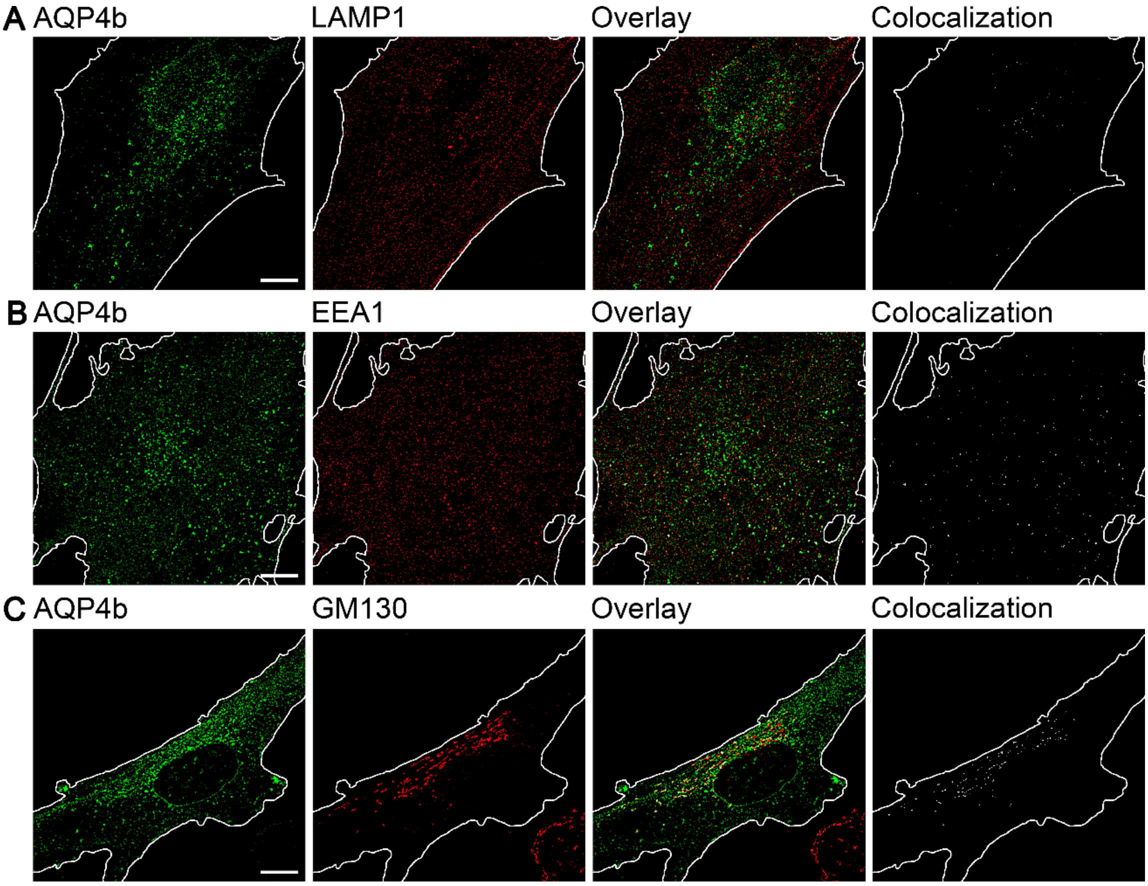


**Supplementary Figure 1**. Colocalization of AQP4b with selected intracellular compartments in astrocytes. Structured illumination microscopy micrographs of rat astrocytes in isoosmotic conditions overexpressing AQP4b immunolabelled with antibodies against (**A**) lysosomal-associated membrane protein 1 (LAMP1), (**B**) early endosome antigen 1 (EEA1) and (**C**) Golgi membrane protein GM130 (second panels). Superimposed signals of the first two columns of panels are represented in the overlay panels and as colocalization masks (white spots). Scale bars (all panels): 10 µm.

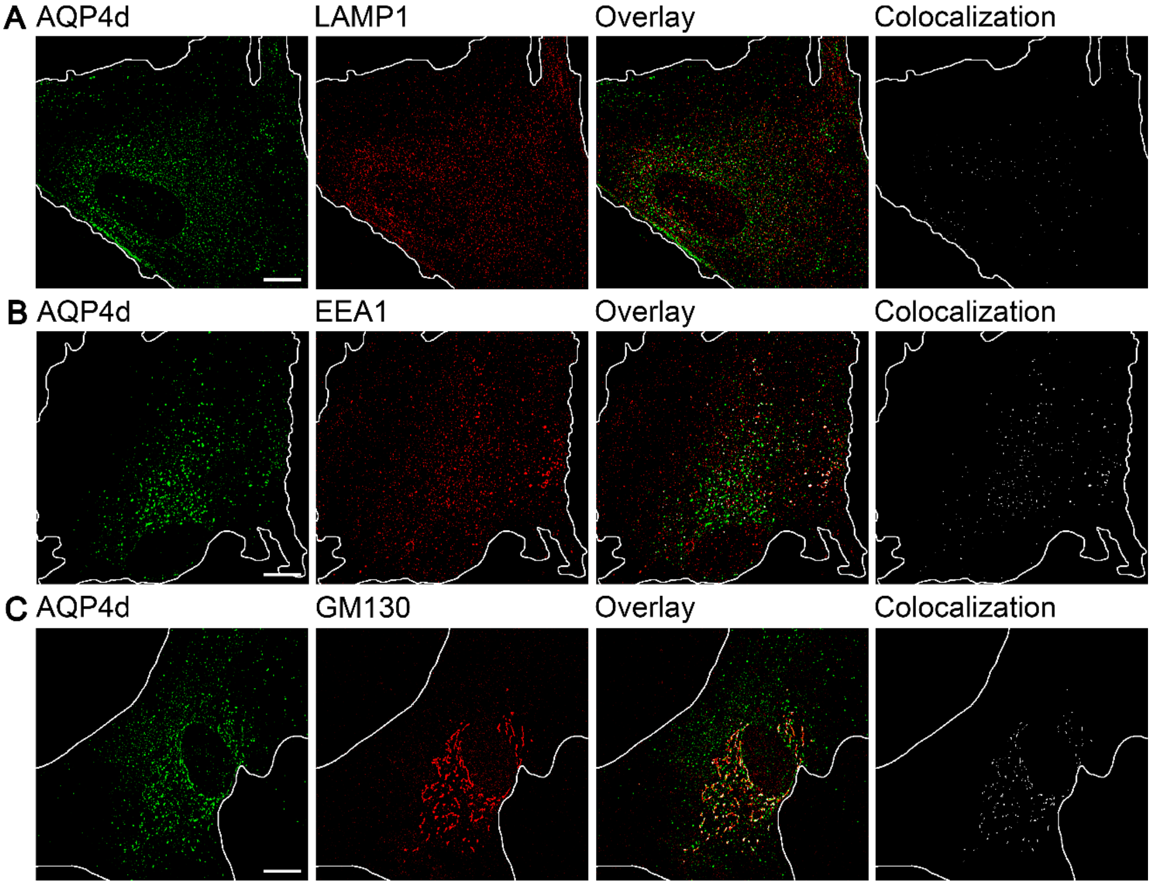


**Supplementary Figure 2**. Colocalization of AQP4d with selected intracellular compartments in astrocytes. Structured illumination microscopy micrographs of rat astrocytes in isoosmotic conditions overexpressing AQP4d immunolabelled with antibodies against (**A**) lysosomal-associated membrane protein 1 (LAMP1), (**B**) early endosome antigen 1 (EEA1) and (C) Golgi membrane protein GM130 (second panels). Superimposed signals of the first two columns of panels are represented in the overlay panels and as colocalization masks (white spots). Scale bars (all panels): 10 μm.
